# Implementation of Mobile-Based Programs for Alcohol Cessation in the Treatment of Alcohol-associated Liver Disease: Protocol for a Type 1 Hybrid Implementation-Effectiveness Trial

**DOI:** 10.2196/94231

**Published:** 2026-05-29

**Authors:** Cecilia He, Jordan Edwards, Linda S Park, Mallory Herzog, Xiang Li, Alice Pulvermacher, Marlon P Mundt, Randall Brown, Jessica Mellinger, Andrew Quanbeck

**Affiliations:** 1 Department of Family Medicine and Community Health University of Wisconsin Madison Madison, WI United States; 2 Department of Internal Medicine, Division of Gastroenterology and Hepatology Henry Ford Health-Michigan State University Detroit, MI United States

**Keywords:** alcohol-associated liver disease, ALD, alcohol use disorder, alcohol use, alcohol cessation, digital health, mobile apps, evidence-based intervention, implementation science

## Abstract

**Background:**

The rising burden of alcohol-associated liver disease (ALD) calls for effective interventions. Alcohol cessation remains the only intervention known to reduce long-term ALD morbidity and mortality. Integrating treatment for alcohol use disorder with medical and hepatology care shows significant promise.

**Objective:**

This study aims to conduct a randomized, controlled, type 1 hybrid implementation-effectiveness trial in patients with ALD to evaluate an evidence-based smartphone app for alcohol use disorder (Connections). The primary aim is to (1) compare the effectiveness of the Connections app plus usual care to the effectiveness of usual care alone on days of alcohol abstinence for patients with ALD and (2) assess the implementation of the Connections app in the ALD population using a multilevel model of system change to determine facilitators and barriers to the successful adoption of the app at the patient, provider, and clinic levels.

**Methods:**

Study procedures were approved by the Institutional Review Board on March 13, 2024. Patients are recruited from General Hepatology and Multidisciplinary Clinics in Wisconsin and Michigan. Participants are randomized to an intervention (Connections + treatment as usual) or no intervention (treatment as usual). During the 6-month enrollment period, participants complete monthly e-surveys measuring alcohol consumption and quarterly e-surveys measuring patient health indicators and behaviors. Patients earn up to US $240 for participation.

**Results:**

This study was funded in September 2023. Recruitment began in June 2024 and is ongoing. As of February 26, 2026, 144 participants have been enrolled in the study. Of these, 65 (45.1%) have completed all the study activities. We aim to recruit 298 participants through 2027, with analysis and results publication estimated in 2028.

**Conclusions:**

Implementation insights highlight the importance of flexibility and strong provider relationships. Challenges include reliance on technology, which limits access for patients with low digital literacy, and the risk of loss to follow-up. This trial is the first fully powered implementation-effectiveness study of a mobile health app for alcohol cessation in ALD, testing a new care model that integrates multidisciplinary expertise.

**International Registered Report Identifier (IRRID):**

DERR1-10.2196/94231

## Introduction

### Background

Alcohol-associated liver disease (ALD) rates have increased markedly over the past 15 years [1,2], becoming the most common indication for liver transplantation in the US and generating the majority of health care and cost burden among all liver diseases [3,4]. ALD encompasses a spectrum of liver disease, including fatty liver, steatohepatitis, cirrhosis, and alcohol-associated hepatitis, which carries a 50% mortality rate at 3-6 months [5]. Nearly all heavy drinkers develop fatty liver, up to 35% develop steatohepatitis, and between 10%-20% develop cirrhosis, the most severe form of ALD [5].

Since the COVID-19 pandemic, unhealthy alcohol consumption patterns have continued to increase in some groups and are associated with a substantial increase in the number of patients presenting with acute alcohol-associated liver injury [6-10]. Drinking levels have remained higher than prepandemic levels, and it is estimated that ALD-related mortality will double by 2040 if nothing is done to address the trend [11].

Despite decades of medical research, alcohol cessation remains the only intervention that is known to substantially decrease long-term ALD morbidity and mortality, and is recognized by the American Association for the Study of Liver Diseases as the only measure known to reverse or prevent the progression of ALD [5]. However, only 10-15% of patients with ALD access treatment targeting unhealthy alcohol use in the first year after their diagnosis, with women being even less likely to access treatment than men [12,13]. Despite these gaps, limited abstinence-based behavioral interventions have been proven to help people with ALD abstain from drinking [14-16], though this evidence is limited in part because so few studies have been conducted exclusively in patients with ALD. Patients with ALD are often excluded from alcohol use disorder (AUD) treatment efficacy studies for reasons such as high mortality, preferences for populations with less complicated conditions, and altered drug pharmacokinetics in patients with advanced ALD [17].

While several randomized studies for alcohol reduction have been conducted in patients with Hepatitis C or HIV and active alcohol use [18-22], only a handful of small randomized clinical trials of behavioral treatments for alcohol cessation have been conducted in patients with alcohol-associated cirrhosis. Of those that have been tested, integrated behavioral treatment alongside medical and hepatology care has consistently shown the greatest impact in reducing alcohol use in patients with ALD [22-27].

Many patients encounter barriers when seeking AUD treatment, making it more challenging to obtain such care [28]. Given the high mortality of patients with ALD who continue drinking, particularly among women with ALD, new evidence-based interventions are needed to leverage hepatology clinic appointments to reach this complex, vulnerable patient population [29-32].

Supplementation of clinical services with evidence-based, mobile technology offers an innovative approach to improving clinical care for patients with ALD. However, very few of these apps have been rigorously tested [33], and none have been designed specifically for or studied in patients with ALD. Given the growing use of telehealth services and the barriers patients with AUD often experience, AUD mobile health (mHealth) treatment apps are uniquely positioned to improve treatment engagement in this population.

### Objectives

The goal of the study, Implementation of Mobile-based Programs for Alcohol Cessation in Treatment of Alcohol-associated Liver Disease (IMPACT-ALD), is to implement and evaluate an evidence-based mHealth smartphone app called “Connections” to support patients with ALD with their liver health and in achieving alcohol cessation.

The primary aim is to (1) compare the effectiveness of the Connections app plus usual care to the effectiveness of usual care alone on days of alcohol abstinence for patients with ALD and (2) assess the implementation of the Connections app in the ALD population using a multilevel model of system change to determine facilitators and barriers to the successful adoption of the app at the patient, provider, and clinic levels. Secondary outcomes will include the impact of the Connections app on health outcomes and changes in health behaviors.

## Methods

### Study Design

The study is a randomized, controlled, type 1 hybrid implementation-effectiveness trial. A hybrid type 1 design is used to test the effects of an intervention while simultaneously gathering information related to implementation [34].

### Study Setting

Recruitment for this study will take place at 2 large tertiary care centers in Wisconsin and Michigan. Each center has both general hepatology clinics as well as integrated, multidisciplinary ALD clinics, which consist of co-located clinicians providing hepatology and addiction and/or mental health care to patients with advanced ALD (cirrhosis and/or acute alcohol-associated hepatitis) [35,36]. Care for patients with ALD in general hepatology clinics is provided by a hepatologist or an advanced practice provider and consists of evidence-based hepatology care with a referral to formal AUD treatment given on a provider-to-provider basis. Recruitment will occur for 3 years from June 2024 through 2027. The study management site will be housed within Wisconsin.

### Eligibility Criteria

Inclusion criteria: to be considered eligible, participants must meet the following criteria:

1. Age 18 years or older
2. Diagnosis of ALD (any stage)
3. Alcohol use within the last 6 months
4. Receiving care at the participating sites’ general hepatology clinic or the multidisciplinary ALD clinic
5. Able to read and write proficiently in English
6. Willing and able to use a smartphone app

Exclusion criteria: participants with the following conditions will be excluded from this study:

1. Actively listed for liver transplant or history of liver transplant before being enrolled in the study. Participants added to a liver transplant list after being enrolled in the study will be allowed to continue their participation
2. In hospice care
3. Has severe cognitive impairment (as described in electronic health record [EHR], including dementia, delirium, and/or unable to maintain cognitive alertness during screening – as determined by study staff)

To engage in the research, all participants will need to have a smartphone to complete baseline, monthly, and quarterly surveys, and if randomized to the intervention arm, to be able to create an account in the Connections app.

### Intervention

Participants will be randomized to one of two groups: intervention (Connections app with treatment as usual) or no intervention (treatment as usual). The intervention group will have access to all the features of the Connections app on their phones for the duration of the study. The Connections app is based on principles of effective care for substance use disorders, such as sustained duration (6-month observation period), peer support, improving coping skills in high-risk situations, assertive outreach, self-monitoring, prompts, and action planning. The theoretical foundation of the Center for Health Enhancement Systems Studies (CHESS) Health is self-determination theory [37], which holds that an individual’s adaptive functioning can be improved if the patient feels (1) competent, (2) related to others, and (3) internally motivated rather than coerced in one’s actions.

The Connections app has both static content (eg, audio-guided relaxation exercises) and interactive features (eg, an alert if a patient receives a response to a discussion group post). The app was designed to serve as a place where participants can find community and support to help manage ALD, learn self-care tips for liver health, coping skills, and alcohol abstinence strategies. The Connections app meets investigational device exemption nonsignificant risk by the US Food and Drug Administration.

### Outcomes

#### Primary Outcomes

The primary outcome is patient alcohol use, which is measured using the patient-reported timeline follow-back (TLFB) calendar method and will be reported as percent days abstinent (Table 1) [38]. Abstinence, number of days drinking, and number of drinks per drinking day will be captured in the patient surveys. The TLFB will be administered once a month for 6 months to all enrolled participants using REDCap (Research Electronic Data Capture; Vanderbilt University), a password-protected electronic data capturing tool hosted at the University of Wisconsin-Madison [39].

**Table 1:** Study outcomes, proposed outcome mediators, and outcome measurements used. Each dimension is measured by a well-validated instrument (Measure) and collected either from a patient survey, CHESS server logs, or from patient and/or staff interviews (Source) at different time points of the study (Timing). The selected measures allow for control of factors that may differ between patients and may impact alcohol use. The implementation process will be assessed using qualitative methods via semistructured patient and staff interviews.

Dimension			Measure		Source		Timing
**Primary effectiveness outcomes**							
	Alcohol use	Timeline follow-back last 30 days (Abstinence, # of days drinking, # of drinks per drinking day)		Patient survey		Monthly	
**Secondary effectiveness outcomes**							
	Quality of life	PROMIS^a^ Global Health [40]		Patient survey		0, 3, 6 months	
	Depression	PHQ-8^b^ [41]		Patient survey		0, 3, 6 months	
	Anxiety	GAD-7^c^ [42]		Patient survey		0, 3, 6 months	
	Insomnia	Insomnia severity index [43]		Patient survey		0, 3, 6 months	
	Health care usage and engagement with alcohol-targeted interventions	Health care usage questionnaire		Patient survey		0, 3, 6 months	
	Drinking motivations	DMQ-A^d^ [44]		Patient survey		0, 3, 6 months	
	Alcohol risk assessment	AUDIT-10^e^ [45]		Patient survey		0, 6 months	
	Pain intensity and interference	PEG^f^ [46]		Patient survey		0, 3, 6 months	
	Relatedness (mediator)	CHESS^g^ Bonding Scale [47]		Patient survey		0, 3, 6 months	
	Competence (mediator)	DTCQ-8A^h^ [48]		Patient survey		0, 3, 6 months	
	Autonomous motivation (mediator)	TSRQ^i^ [49]		Patient survey		0, 3, 6 months	
	Primary moderator variables (sex, rural and/or nonrural, clinic site)	Sex, rural and/or nonrural, clinic site (multidisciplinary ALD^j^ vs general hepatology)		Patient survey		0 months	
	Secondary moderator variables (race, ethnicity, education level, age, marital status, ALD severity)	Adapted upstream risks screening tool (URST)		Patient survey		0 months	
**Implementation measures**							
	CHESS use (patients)	Number of days used; number of pages viewed		CHESS server logs		Continuous	
	Qualitative assessment	Stakeholder assessments of the implementation process		Patient and/or staff interviews		Years 3-4	

^a^PROMIS: Patient-Reported Outcomes Measurement Information System.

^b^PHQ-8: Patient Health Questionnaire-8.

^c^GAD-7: General Anxiety Disorder-7.

^d^DMQ-A: Drinking Motives Questionnaire for Adults.

^e^AUDIT-10: Alcohol Use Disorders Identification Test.

^f^PEG: Pain, Enjoyment, and General Activity 3-item scale.

^g^CHESS: Center for Health Enhancement Systems Studies.

^h^DTCQ-8A: Drug-Taking Confidence Questionnaire.

^i^TSRQ: Treatment Self-Regulation Questionnaire.

^j^ALD: alcohol-associated liver disease.

#### Secondary Outcomes

The secondary outcomes include quality of life, depression, anxiety, insomnia, health care usage, engagement with alcohol-targeted interventions, drinking motivations, alcohol risk assessment, and pain intensity and interference (Table 1). Quality of life will be measured using a 10-question Patient-Reported Outcomes Measurement Information System Global Health (PROMIS Global Health) [40], a questionnaire that evaluates and monitors physical, mental, and social health. Depression will be measured using the Patient Health Questionnaire (PHQ-8) [41], a tool used for screening, diagnosing, monitoring, and measuring the severity of depression. Patient anxiety will be measured using the General Anxiety Disorder-7 (GAD-7) [42], and insomnia will be measured using the insomnia severity index [43]. Engagement with alcohol-targeted interventions will be measured using participation in AUD treatment outside of the clinic, such as specialty outpatient treatment, peer support, or Alcohol or Other Drug Abuse treatment, as reported in the health care usage survey. Motivation for drinking will be measured using the Drinking Motives Questionnaire for Adults (DMQ-A) [44], and alcohol use risk will be measured with the Alcohol Use Disorders Identification Test (AUDIT-10) [45]. Pain intensity and functional interference will be measured using the Pain, Enjoyment, and General Activity 3-item scale (PEG) [46]. Survey measures will be administered at baseline, 3 months, and 6 months.

#### Mediators

Mediators include 3 fundamental psychological needs specified by self-determination theory [37]. Relatedness will be measured by the CHESS Bonding Scale [47], a 5-item scale used to capture bonding with other patients with ALD. Competence will be measured by the Drug-Taking Confidence Questionnaire (DTCQ-8A) [48], a tool used to measure a person’s confidence in their abilities to cope in situations that are high-risk for substance use, or relapse potential. Autonomous motivation will be measured by the modified Treatment Self-Regulation Questionnaire (TSRQ) [49], which uses a 7-point Likert scale to respond to 5 items assessing the degree to which a person’s motivation for healthy behavior is autonomous.

#### Power and Sample Size

The analysis assumes an intra-individual correlation coefficient of ρ=0.10 in alcohol abstinence days in the longitudinal mixed-effects model. With 7.5% loss to follow-up and a moderate effect size estimate of 0.3σ (using published effect sizes from prior CHESS platform studies) [50,51], the planned sample size of 298 participants (149 per randomization group) will have power ≥80% to detect a meaningful difference between randomization groups for the primary outcome of alcohol abstinent days.

The projected randomization of 298 participants is expected to be met due to the estimated recruitment pool of approximately 2361 patients with ALD across both sites. Of this total population, it is expected that approximately 690 patients will be part of the multidisciplinary ALD clinics. The randomization goal is less than 13% of the total estimated eligible population, which is likely a feasible recruitment rate based on prior studies led by members of the research team [52]. This feasibility is further supported by the recent pilot research recruitment rate of 11 out of 20 (55%) patients with ALD approached [53].

#### Participant Recruitment

Patients presenting to both general hepatology and multidisciplinary ALD clinics at the recruitment sites are contacted by research coordinators during or after their clinic visit. Several recruitment strategies are used across both sites:

Clinic flyers: Institution Review Board (IRB)–approved study flyers are posted in waiting rooms and patient care rooms with study details. Flyers will include a QR code for patients to scan that links to a screening survey to determine eligibility. Study phone numbers and email addresses are included if potential participants have questions.

Scheduled patient appointments: study team members will use automated recruitment algorithms that search patient EHRs to identify potentially eligible participants and their scheduled appointments. Study coordinators will be available in clinic during these appointments to approach patients.

Provider referrals: potentially eligible patients can be identified by providers when reviewing their clinic appointments for the week, including patients who may not appear on the automated recruitment algorithm list. Providers may recommend the study to potentially eligible patients and inform the study staff if a patient is willing to hear more about the study.

Participants may receive up to US $240 for the surveys completed (Table 2).

**Table 2:** Participant survey payment distribution. Participants are compensated for completing surveys throughout the 6-month study. Payments are distributed throughout the six months as each survey is completed.

Survey	Amount (US $)	Payment timing
Screener	10	After screener completion
Baseline	40	After randomization
Monthly	10	Every month during 6-month study period
3-month quarterly	30	After survey completion
6-month quarterly	100	After survey completion

### Participant Timeline

Participants’ involvement in the study can be divided into three distinct phases: (1) a pre-enrollment period encompassing screening completion and reviewing informed consent; (2) an onboarding period consisting of completion of baseline surveys, randomization, and downloading Connections if assigned to the intervention; and (3) a data collection period consisting of completing monthly surveys for 6 months. The pre-enrollment and onboarding phases can be completed in-person, virtually, or over the phone, and may take up to 60 days to complete. Once enrolled, a participant will be engaged in the study for 6 months.

### Pre-Enrollment

#### Screening Survey

Patients that express interest are asked to complete a screening survey. If eligible, a study team member will confirm eligibility by reviewing the patient’s EHR. If ineligible, the patient will be notified and will receive compensation for completing the screener if they have provided necessary contact or payment information.

#### Health Record Review

Patients that screen as eligible through the survey will receive follow-up by a research team member. Study staff will review the patient’s EHR to confirm diagnosis of ALD, specify the type of ALD (if given), and confirm that the patient does not meet any of the exclusion criteria. Once confirmed, the patient will be notified of their eligibility status.

#### Informed Consent

Eligible participants will review the informed consent information with a study team member, either in-person during the clinic visit or virtually (eg, Zoom [Zoom Communications, Inc]). Signatures are obtained on paper copies in-person or signed electronically using a HIPAA (Health Insurance Portability and Accountability Act)-compliant platform. The patient will receive a signed copy for their records. If a patient declines to participate, reasons for refusal will be documented, and the study team will not initiate further contact with the individual.

### Onboarding

#### Baseline Surveys

Once participants consent to the study, they are prompted to complete baseline surveys and a TLFB calendar survey. Once all surveys are completed, the participant can be randomized to a study arm. Participants who complete the screening survey, review, and sign the informed consent with study staff have up to 60 days to complete the baseline surveys and TLFB. If the participant exceeds 60 days, they are marked as “off-study,” and the reason for exclusion (Subject declined to participate prior to randomization) is documented accordingly.

#### Randomization

This study uses a 1:1 block randomization plan for participant assignments to the intervention arm (n=149) versus no intervention arm (n=149), stratified by recruitment site (Wisconsin versus Michigan) and recruitment clinic type (general hepatology clinic versus multidisciplinary ALD clinic). The randomization tool within REDCap will be used for the randomization process, providing study arm assignments based on a predetermined allocation table programmed with the site and clinic type stratifications. To prepare for randomization, research staff will verify that informed consent has been signed and that all baseline surveys, including the TLFB, have been completed. During randomization, the research staff member opens REDCap, enters the current date, and initiates the REDCap randomization process. REDCap then assigns the participant to a study arm and notifies the staff member of the assignment outcome. This can take place either in-person during the clinic visit, over the phone, or in a virtual meeting. If the patient is assigned to the intervention group, study staff will assist them in downloading the Connections app and will guide them through its features. Participants using Connections are encouraged to create an alias name to protect their identity.

After randomization, a participant is considered enrolled in the study. They will remain in the study for 6 months following enrollment. During this time, they will complete monthly and quarterly surveys until the end of their study period. Participants may withdraw their consent at any time throughout the 6-month period after enrollment, at which time the study team will document their request and deactivate their account. The participant will not receive any further communication from the study.

### Data Collection, Management, and Analysis

#### Quantitative Data Collection

The quantitative data collected in this study will come from multiple sources, including monthly and quarterly surveys, and Connections app use. Patients who screen eligible on the pre-screening survey (Day 0) will have up to 60 days to complete baseline surveys and enroll in the study. After joining, participants must complete monthly surveys within 14 days of the first reminder for the six-month study period. The study also includes two follow-ups: at 3-months (surveys due 80 to 110 days after joining) and 6-months (surveys due 170 to 200 days after joining).

#### Monthly Surveys

Participants are prompted via text or email to complete monthly surveys to track their alcohol consumption for 6 months after randomization. A TLFB survey asks participants to report the number of standard drinks consumed in the last 30 days, with the option to specify the event (vacation, birthday, holiday, wedding, sports, party, travel, stress, or other). Participants are sent up to 3 reminders to complete the monthly TLFB and have a total of 14 days to complete the survey.

#### Baseline and Quarterly Surveys

During months 0 (baseline), 3, and 6, participants are prompted to complete a series of surveys sent as a text or email. Participants have a 30-day window to complete the surveys for compensation.

#### Connections Use

Connections app usage will be recorded automatically in server log files and collected continuously during the 6-month intervention. Use is defined as patients accessing Connections and going beyond the home page. The study team will examine the number of days on which patients use the app and the mean number of pages used beyond the home page.

#### Qualitative Data Collection

To explore barriers and facilitators to successful implementation of the Connections app at the patient, provider, and clinic levels, qualitative interviews will be conducted with key stakeholders about their perspectives on the implementation process. A qualitative researcher experienced in mHealth apps will conduct interviews. Semistructured interview guides will include questions based on the domains of the National Academy of Engineering’s (2005) multilevel model of system changes, as well as more open-ended questions designed to probe the participants’ own experiences and opinions [54]. Data collection and analysis for assessing the implementability of the Connections app will happen in years 3-5 at the conclusion of the active intervention.

A total of 30 patients from both Wisconsin and Michigan will be interviewed: 15 who used the Connections app extensively during the intervention (ie, from the top quartile based on overall use) and 15 who did not (from the bottom quartile). Participants will be purposively sampled from these strata to ensure a balanced sample for sex and multidisciplinary ALD clinic vs general hepatology-only clinic attendance, as these are 2 hypothesized key moderators of effect. Patients will be asked about the ease of use and acceptability of the content, features, and functioning of the Connections app, their overall experience with learning about the app, and suggestions for improving how the clinic introduces patients to the app and provides ongoing support for its use.

Additional interviews will be conducted with clinic staff and study coordinators to gather information about the implementation process. Realms for exploration will include the following: (1) What types of workflow and process changes were needed to maximize the adoption of the Connections app in the clinical setting? (2) What helped staff to implement these changes? Providers and staff will also be asked about their observations of any differences in app adoption and use between patient categories or characteristics, and about theoretical challenges with implementing an app like this in clinic.

The research team will debrief after early interviews and make any needed modifications to the interview guides. Interviews will be audio-recorded via secure web platform such as Zoom or similar (version 5.11.3).

### Data Analysis

#### Primary Analysis

To test the primary aim, alcohol consumption outcomes will be compared between the usual care condition and the usual care plus the Connections app condition, stratified by site and clinic type, and adjusting for baseline abstinence and risky drinking (AUDIT-10 score). The analysis will use an intent-to-treat paradigm and use longitudinal linear mixed-effects models. The analyses will test a group-by-time interaction on the outcomes. If the interaction is not significant, a main group effect will be examined. The analysis assesses direct treatment effects on patient outcomes over time. The research team will construct a longitudinal model of our outcome measures at baseline and at 3 and 6 months after randomization. To address potential incomplete data from individual subjects, the research team will also conduct a mixed-model analysis of repeated measures based on the general linear model with an assessment of various covariance structures (compound symmetric, autoregressive order one, and unstructured). Covariance structure selection will be based on Akaike’s information criterion and Schwarz’s Bayesian criterion [55,56]. Pairwise comparisons between treatment groups and specific treatment time contrast in the mixed model will be conducted to respond to between-group effects and time-based effects. To address issues of incomplete data, the research team plans to estimate treatment effects according to four approaches: intention-to-treat, as-treated, per-protocol, and complier-average causal effect. Secondary analyses (as-treated, per-protocol, CACE) will only be formally evaluated if the primary intention-to-treat outcome demonstrates a statistically significant effect. Details of these approaches are outlined in Little and Yau [57] and Jo [58]. Missing data patterns (eg, possible dropouts) will be established from the longitudinal data and will be used to adjust the longitudinal intervention analysis according to pattern-mixture modeling. The research team will also examine additional secondary hypotheses (Table 3).

**Table 3:** Summary of secondary study hypotheses. Predicted relationships between intervention engagement, participant characteristics (sex and location), and clinical outcomes, including proposed mediating mechanisms, to be examined during quantitative analysis.

Variable	Description of hypothesis
Intervention group	Patients in the intervention group will show improvements in secondary health outcomes compared to patients in the control group (depression, anxiety, insomnia, liver health, and engagement in alcohol-related interventions and recovery activities).
Sex as a biological variable	Sex will moderate effects of Connections. Specifically, women receiving Connections will have greater improvements in outcomes than men.
Rural and/or nonrural locations	Living in rural versus nonrural locations will moderate effects of Connections. Specifically, those in rural areas will have greater improvements in outcomes than those in nonrural areas as defined by rural-urban continuum code.
Clinic type	Attendance at the ALD^a^ clinic offers attention to recovery and formal alcohol use disorder treatment as part of ALD treatment, potentially enhancing motivation and providing a foundation for deriving greater benefit from Connections. We hypothesize that those attending multidisciplinary ALD clinics will demonstrate greater benefit from Connections than those attending hepatology clinics.
Competence, relatedness, and autonomy as mediators	Effects of Connections on outcomes will be mediated by competence, relatedness, and autonomous motivation. We will test indirect effects of Connections on abstinence, indirect effects, and percent of the total effect mediated. Mediation analyses will only be undertaken if the main effect is statistically significant.
CHESS^b^ app use	Increased use of Connections will be associated with greater improvements in abstinence days. Greater use of Connections may reflect patients’ motivation for recovery and provide more opportunities to engage with recovery support.

^a^ALD: alcohol-associated liver disease.

^b^CHESS: Center for Health Enhancement Systems Studies.

#### Moderator Analysis

To guide future app refinements and deployment strategies, moderation analyses will assess which patient characteristics are associated with deriving greater benefit from using Connections. For each proposed moderator, a linear regression model will be built to predict abstinence days at 6 months, controlling for abstinence days at baseline. An interaction term will be added to the model which crosses the proposed moderator with intervention group. Significant interaction effects will be decomposed to understand the differences between subgroups. We will examine potential moderation by sex, race, ethnicity, age, stage of ALD, comorbidities, marital status, education, AUD treatment engagement, and AUD treatment confidence, intention, readiness, and importance rulers, as well as testing directional hypotheses for the proposed moderators. Notably, we predict that sex will largely moderate effects of Connections, with women benefiting more than men in the intervention arm. Moderator analyses are exploratory and are considered hypothesis-generating rather than confirmatory.

#### Qualitative Data Analysis

All interviews will be audio-recorded and transcribed verbatim by a HIPAA-secure transcription service that has a Business Associate Agreement with the study team. Audio-recorded data of interviews will be stored in a department secure box folder accessed via password-protected computers, then uploaded for analysis via qualitative data analysis software (Dedoose, version 10.0.25; SocioCultural Research Consultants; a cross-platform app for qualitative and mixed methods research). Data analysis will take place concurrently with data collection and will proceed iteratively. In the first stage of the analysis, transcripts will be coded using both directed content analysis and traditional content analysis. The initial focus will be on populating a priori concepts and on inductively developing new concepts. Subsequent stages of the analysis will see several additional coding passes and memo writing and will aim to develop higher-order categories, explicate the mechanisms linking these categories, and explore how identified conditions (“facilitators and barriers”) affect these mechanisms. In addition, comparative analyses will look for ways in which patient characteristics, provider and/or staff roles, and clinic type appear to affect app adoption and use.

### Data Monitoring

All electronic study data will be stored in secure, password-protected servers. No data collected by the app will be entered into patients’ electronic medical records or affect the legal medical record. All participants are assigned a study ID number, and all data will be deidentified before exporting for statistical analyses. Subjects randomized to the Connections app will be encouraged to select an anonymous username and password for the app. All Connections app data use will be collected using only the anonymous usernames. Any hard copy-identifying information will be stored securely in a locked cabinet. When all study activities are complete, all identifiable information will be destroyed; only the deidentified codes will remain.

### Ethical Considerations

This protocol was initially approved by the Health Sciences Minimal Risk IRB (2024-0130) with subsequent annual reviews. Informed consent was reviewed and obtained from all patients prior to study enrollment. This consent and IRB approval cover secondary analysis using existing data collected. Additional consent will be obtained from participants for qualitative patient interviews prior to collection. Participants are allowed to withdraw from the study at any point by notifying the research study team, which is then documented in the database. All staff involved in the study will remain compliant with their institution’s Human Subjects Protection and the HIPAA Privacy Act. Participants receive up to US $240 for joining the study.

## Results

This study was funded in September 2023 with IRB approval in March 2024. Recruitment and data collection began in June 2024 in Wisconsin and June 2025 in Michigan. As of February 26, 2026, 144 participants have been enrolled in the study (Wisconsin: n=97, Michigan: n=47). Of these, 73 (50.7%) are currently in their 6-month study period, 65 (45.1%) have completed all study activities, and 6 (4.2%) were removed from the study due to participant withdrawal or death (unrelated to the study). We aim to recruit up to 298 participants through 2027, with analysis and results publication estimated in 2028.

## Discussion

### Anticipated Findings

The study’s primary hypothesis anticipates that patients in the intervention group will have more abstinent days compared to patients in the usual-care group. Additionally, we anticipate the secondary hypotheses as outlined in Table 3. This includes patients in the intervention group showing greater improvement in secondary health outcomes compared to patients in the usual-care group, women in the intervention group benefiting more than men, patients in rural areas having greater improvements than patients living in nonrural areas, patients attending multidisciplinary ALD clinics receiving greater benefit from the intervention than those attending general hepatology clinics, and the association of increased Connections app use with overall greater improvements. Note that these secondary outcomes are exploratory.

### Comparison With Prior Work

Previous studies have shown that appropriate mental health, substance use, and medication interventions can improve liver-related outcomes [12]. Although a liver disease diagnosis can be a powerful motivator to abstain from alcohol use and engage in treatment, there are many barriers that prevent patients with ALD from engaging in AUD treatment [28]. Attitudinal barriers, such as stigma, and logistical barriers, such as transportation issues or financial barriers, further prevent patients from seeking care. Thus, use of a mHealth app may help overcome the unique barriers to AUD treatment that patients with ALD face.

We conducted a mixed-methods pilot evaluation of a version of the app used in IMPACT-ALD in a sample of patients with ALD prior to submission of the grant that funded the study. In that pilot study, we found that patients are motivated to stop drinking after getting an ALD diagnosis and open to using a digital recovery tool [53].

In another pilot randomized trial of an mHealth app with patients with ALD, use of the app improved AUD treatment engagement rates (27.3% vs 13.3%, odds ratio [OR] 2.3, 95% CI 0.61-8.76) and resulted in a greater proportion of subjects with a reduced drinking risk level (OR 2.25, 95% CI 0.51-9.97) [59]. While this mHealth app structure differs from that of Connections, the pilot data support the feasibility and acceptability of alcohol use-focused mHealth apps in the ALD population.

### Strengths

This study has notable strengths. To our knowledge, this trial is the first fully powered implementation-effectiveness study of a mHealth app for alcohol cessation in ALD, testing a new care model that integrates experts in systems engineering, medicine, digital health, and implementation science.

### Challenges

The implementation of a mHealth system presents several challenges. First, although mHealth approaches can increase overall accessibility to patients with ALD, they rely heavily on technology use and may be challenging for populations with low digital literacy. After enrollment, all components of the study require technology use, including completing monthly and quarterly surveys or exploring the CHESS Connections app if randomized to the intervention arm. Participants with low digital literacy may be less likely to check their emails or app notifications, which can lead to lower response rates, or they may be less likely to explore the intervention app. To address this, research staff have implemented frequent survey reminders to encourage study engagement. Second, while recruiting patients in-person during a clinic visit is preferred, it is time-consuming to enroll a patient from screening through randomization, particularly for patients to complete the baseline surveys, which could vary in length depending on the patient’s literacy skills and comfort level with technology. This often deters patients from completing enrollment during the clinic visit, leading to challenges such as missed virtual meetings to finish enrollment and patients that are lost-to-follow-up, even with multiple researcher follow-up attempts. Initially, patients used the MyCap app (Vanderbilt University)—a mobile, patient-facing version of REDCap—to complete baseline surveys and would download and create an account during onboarding. However, we learned that this lengthened the enrollment process substantially and have since eliminated the use of MyCap, opting for surveys to be sent through email or text instead. Another proposed challenge is the reliance on patient self-reporting of alcohol use, which introduces the possibility of underreporting or recall bias due to the 30-day recall period in the monthly TLFB surveys. Finally, while disparities for racial and ethnic minority patients are evident across the health care system, the higher rates of ALD among White patients parallels the patient panel in the 4 recruitment clinics in this study. This may limit the diversity of the study sample and decrease the ability to recruit any significant number of racial and ethnic minority patients into the study. To partially mitigate this concern, we will oversample patients from underrepresented groups during the qualitative portion of the study.

### Future Directions

Successful implementation of the Connections app for patients with ALD would expand AUD treatment access, providing critically needed alcohol cessation tools to those most vulnerable to disease progression due to lack of engagement in AUD care. Because alcohol cessation is crucial to long-term survival, innovative methods to connect patients with ALD to AUD treatment, such as apps similar to Connections, hold promise to close the evidence-to-practice gap for patients with ALD and to ultimately reduce ALD mortality. As noted, qualitative interviews will be conducted with key stakeholders, including research participants, clinic staff, and the research study team, to explore barriers and facilitators for successful implementation of a digital health tool.

## Data Availability

The data sets generated during this study are available on the Clinical Trials site (#NCT06305624) and NIMH Data Archive site (NDA Collection 5034).

## Appendix

Multimedia Appendix 1 Peer review report from the National Institute on Alcohol Abuse and Alcoholism Special Emphasis Panel (National Institutes of Health, USA).

## Abbreviations

AUDIT-10: Alcohol Use Disorders Identification Test
ALD: alcohol-associated liver disease
AUD: alcohol use disorder
CHESS: Center for Health Enhancement Systems Studies
DMQ-A: Drinking Motives Questionnaire for Adults
DTCQ-8A: Drug-Taking Confidence Questionnaire
EHR: electronic health record
GAD-7: General Anxiety Disorder-7
HIPAA: Health Insurance Portability and Accountability Act
IMPACT-ALD: Implementation of Mobile-based Programs for Alcohol Cessation in Treatment of Alcohol-associated Liver Disease
IRB: Institutional Review Board
mHealth: mobile health
OR: odds ratio
PEG: Pain, Enjoyment, and General Activity 3-item scale
PHQ-8: Patient Health Questionnaire-8
PROMIS: Patient-Reported Outcomes Measurement Information System
REDCap: Research Electronic Data Capture
TLFB: timeline follow-back
TSRQ: Treatment Self-Regulation Questionnaire
