# Supplementary material for: Implementation of Mobile-Based Programs for Alcohol Cessation in the Treatment of Alcohol-associated Liver Disease: Protocol for a Type 1 Hybrid Implementation-Effectiveness Trial
Source: JMIR Res Protoc. 2026 May 29;15:e94231. doi: 10.2196/94231 (PMC13263654; doi:10.2196/94231)
Supplement: Multimedia Appendix 1 [file resprot_v15i1e94231_app1.pdf]

Organization: UNIVERSITY OF WISCONSIN-MADISON

Project Title: Testing an mHealth System for Integrating Alcohol Use Treatment with Hepatology Care for Patients with Alcohol-associated Liver Disease

TABLE OF CONTENTS

Summary Statement (Peer review report) .... 2 – 13

Response Letter to Reviewer Comments 14

1R01AA030470-01A1 Quanbeck, Andrew

PROTECTION OF HUMAN SUBJECTS UNACCEPTABLE

RESUME AND SUMMARY OF DISCUSSION:

This resubmitted application seeks to test the effectiveness of a proven smartphone application for alcohol cessation among patients with alcohol-associated liver disease (ALD).There is a need to develop new interventions for individuals suffering from ALD. Treatment for ALD is a major public health priority, as this disease is growing, especially among young people. There is an acceptable Multiple Principal Investigator leadership plan in place that details the responsibilities of each Principal Investigator for their respective components of the project. The application is conceptually innovative in proposing a combination of a digital health intervention with self-monitoring tools. The applicant has been partially responsive to prior critiques, but some concerns remain in the experimental approach, which reduced the overall enthusiasm for this proposal. Since the majority of the study's population is largely white, applicants would not be able to generalize results nor implement interventions in target populations. The preliminary data provided is insufficient to assess the feasibility of the proposed experimental plan. The eligibility criteria are currently too vague as there is lack of information about the type of patients that will be included in the study, patients with a narrow period of time of alcohol use will be recruited, and there is a risk of decompensation due to the possibility of participants have a wide spectrum of ALD. There were concerns about the overly ambitious experimental plan. Applicants appear to underestimate the complexity of working with a huge sample size that needs to be followed for more than six months and the possibility of dropouts, which has not been properly addressed. The research team is quite large and there is fair amount of redundancy in expertise and roles. There were concerns about the reasoning behind the sample size justification as the power calculation appear to be based on generic estimates. The additional weaknesses stated in the critiques also diminished the overall level of enthusiasm for this application.

DESCRIPTION (provided by applicant):

Alcohol-associated liver disease (ALD) rates have risen markedly over the past 15 years, becoming the most common indication for liver transplantation in the U.S. and generating the majority of healthcare and cost burden among all liver diseases. With the COVID pandemic, these trends have worsened, and it is estimated that, if nothing is done to stem the tide, ALD-related mortality will double by 2040. Despite decades of medical research, alcohol cessation remains the only intervention that substantially decreases long-term ALD morbidity and mortality. However, only 10-15% of ALD patients access alcohol use treatment in the first year after their diagnosis, with women even less likely to access treatment compared to men. Despite these gaps, surprisingly few behavioral interventions for alcohol cessation have been tested in ALD patients, and, of those that have been tested, integrated alcohol cessation treatment alongside medical and hepatology care has had the most impact at reducing alcohol use. To address these critical research gaps, the investigators will perform a randomized, controlled, Type 1 hybrid implementation-effectiveness trial in a population of ALD patients to evaluate the effectiveness of CHESS Health Connections (a smartphone app for alcohol cessation shown to significantly reduce risky drinking in a prior randomized clinical trial of patients with severe alcohol use disorder). Patients will be enrolled in both general hepatology and multidisciplinary ALD clinics (which include integrated alcohol use treatment professionals alongside hepatology providers) at two large tertiary care centers (University of Wisconsin and University of Michigan). Aim 1 will compare the effectiveness of CHESS plus usual care (n=180) versus usual care (n=181) on days of abstinence over 6 months. Aim 2 will assess implementation of CHESS through qualitative interviews of key patient, provider, and clinic-level stakeholders using the Replicating Effective Programs framework and implementation costs. Our secondary/exploratory analyses will examine intervention effects on health outcomes including depression, anxiety, insomnia, AUD treatment engagement, and liver health. We will examine key moderators (age, sex, rurality, presence of formal AUD treatment, and stage of ALD) and mediators (relatedness, competence, autonomous motivation) on outcomes. We will also examine

the impact of the CHESS app on measures of chronic liver impairment using the Model for End-Stage Liver Disease-Sodium score. This study will build on over a decade of work in adapting and using CHESS in various populations with alcohol use disorder. This study is highly innovative in three ways: 1) it is the first fully powered effectiveness trial of a proven smartphone app for alcohol cessation in ALD patients, 2) it will test a new model of care for delivering alcohol cessation treatment to ALD patients, and 3) it will utilize a novel collaboration of systems engineering and medical researchers with expertise in ALD, digital health, and implementation science. If successful, this study holds promise to provide critically needed alcohol use resources to ALD patients and potentially set a new standard of care.

PUBLIC HEALTH RELEVANCE

Project Narrative Rising rates of alcohol-associated liver disease are a public health emergency in the United States, and though alcohol cessation saves patients' lives, few patients have access to evidence-based alcohol interventions. Smartphone applications hold potential to improve outcomes for patients with alcohol-associated liver disease by overcoming barriers to evidence-based alcohol treatment. The investigators will perform a randomized controlled trial testing the effect of a proven smartphone application for alcohol cessation (called CHESS Health Connections app) on alcohol cessation among patients with alcohol-associated liver disease at two medical centers in Michigan and Wisconsin.

CRITIQUE 1

Significance: 2
Investigator(s): 1
Innovation: 2
Approach: 4
Environment: 1

Overall Impact:

This revised application proposes a randomized controlled trial of the CHESS smartphone app to reduce alcohol use among patients with alcohol-associated liver disease (ALD), in addition to assessing implementation via a combination of qualitative data and cost effectiveness analyses. The application has many notable strengths. The investigators are addressing a significant public health problem, given the recent increase in ALD and lack of data on intervention strategies. The CHESS intervention has a growing evidence base and the application now includes pilot data to support feasibility among people with ALD. This is an experienced research team led by PI Dr. Andrew Quanbeck as well as MPIs Dr. Randall Brown and Dr. Jessica Mellinger, who have complementary expertise and established clinical connections in two relevant sites. The study is innovative in that it applies the CHESS intervention to people with ALD. The study uses validated measures for primary and secondary outcomes, and includes well-justified mediation and moderation analyses, in addition to analysis of cost effectiveness which will be led by Dr. Mundt. Inclusion of substantial numbers of women will allow the team to examine sex differences. Pilot data are now included although the sample size was small. Some concerns in the approach were noted regarding sample homogeneity as primarily white, which may impact generalizability, inclusion of abstinent patients in the sample, lack of usual care measurement, and lack of clarity regarding some of the qualitative measures and how these will inform implementation. These concerns were considered minor to moderate. Overall, this is a strong revised application examining an important intervention approach in a high-priority patient population and is likely to have a substantial scientific impact.

1. Significance:

Strengths

- Treatment for ALD is a major public health priority, with recent increases in prevalence among women and younger adults during the COVID-19 pandemic as well as growing mortality. ALD also is costly and burdensome to health care systems.
- Few patients with ALD access alcohol treatment even after ALD has been identified and diagnosed, and interventions have received little attention relative to the prevalence and impact of ALD in the US.
- The study extends the existing evidence base of the CHESS intervention, a digital approach to alcohol treatment that integrates app-based self-management tools as well as peer support.
- Secondary outcomes include conditions common among patients with ALD such as depression, anxiety, and insomnia.
- The application now includes pilot feasibility data, which supports the potential feasibility as well as significance of the research.
- The study proposes to test a scalable intervention in the context of liver disease treatment settings. If the intervention is effective it has the potential to expand alcohol treatment options, including for ALD patients living in rural settings and those who might otherwise have trouble accessing alcohol treatment.
- The study examines implementation as well as intervention efficacy.
- Intervention costs and health care utilization outcomes will be examined.

Weaknesses

- Pilot data, while valuable in supporting feasibility of the intervention, were limited to a relatively small sample of 11 participants over the course of 4 weeks.

2. Investigator(s):

Strengths

- The study is led by PI Dr. Andrew Quanbeck as well as MPIs Dr. Randall Brown and Dr. Jessica Mellinger. This is an established research team with prior experience in digital health interventions, including clinical trials using CHESS.
- The investigators have complementary strengths in alcohol interventions, hepatology, digital health, qualitative methods, and cost effectiveness.

Weaknesses

- None noted.

3. Innovation:

Strengths

- Few prior studies of behavioral or digital health interventions have been conducted among patients with ALD.
- The intervention combines self-monitoring tools with peer support.
- Study design includes implementation as well as effectiveness outcomes, and also includes cost analysis.
- The intervention study is integrated into ALD treatment settings, including one that offers AUD treatment, in a stratified design.

Weaknesses

- The multidisciplinary technological and health care expertise of the team is a strength of the proposal but is not an innovation per se.

4. Approach:

Strengths

- Hybrid Type 1 trial will examine effectiveness as well as implementation.
- The study sites are highly familiar to the research team, and prior work has been conducted in these clinics, which enhances feasibility and likelihood of success in conducting the trial
- Pilot data on feasibility on using CHESS in the target population are now included, supporting the overall study approach and the willingness of patients to enroll in the trial and to complete at least the initial follow-up assessments.
- Substantial planned enrollment of women will allow investigators to examine sex differences in intervention outcomes.
- The study includes validated measures for primary and secondary outcomes.
- Reasonable choice of mediation and moderation analyses that examine effects of app engagement and potential variability in intervention effects for key sociodemographic groups.

Weaknesses

- Eligibility is based on any use of alcohol in the prior year, while outcomes are measured based on use in the prior 90 days. It seems possible that some patients would be eligible for the trial yet already have achieved a positive score on the main study outcome at baseline, which could limit the ability of the efficacy component of the study to determine efficacy although adjustment for baseline abstinence is proposed. It's also not clear what patients who fall into this category will be told about participation, e.g., why they should consider enrolling if they have already been abstinent for 3-12 months. This latter point could potentially be addressed by highlighting relapse prevention-focused intervention components.
- Usual care as it relates to alcohol use is not well described, and it is not clear if usual care components interventions will be measured over the course of the trial in either of the two study sites. For example, it would be valuable to know how much alcohol treatment that patients receive in-clinic, whether patients discuss use of the app with their usual care providers over the course of the trial, and whether there are differences between arms. Such measurement should be considered in order to better understand the additional contribution of the CHESS app, especially since one of the study sites already provides integrated alcohol and mental health treatment for patients with ALD.
- Largely white ALD study population (85%) in the study clinics could limit generalizability to race/ethnic groups, although a plan is described to maximize minority outreach and recruitment.
- Proposed qualitative patient interview questions to assess implementation seem reasonable. However, those proposed for staff (e.g., "What kinds of workflow and process changes were needed to maximize the adoption of CHESS in the clinical setting?" seem to presuppose that the CHESS intervention will have been made broadly available in the clinics rather than confined to patients enrolled in an RCT. In the description of the study procedures, it seems that only the study coordinator will be assisting participants with downloading and use the app.

5. Environment:

Strengths

- Strong environment at the University of Wisconsin Departments of Family Medicine & Community Health, Nursing and Medicine, as well as University of Michigan Medicine and hepatology care clinics.
- Established collaborative relationship with CHESS Health.

Weaknesses

- None noted.

Study Timeline:

Strengths

- Acceptable overall timeline for completing the study activities.

Weaknesses

- None noted.

Protections for Human Subjects

Acceptable Risks and/or Adequate Protections

Data and Safety Monitoring Plan (Applicable for Clinical Trials Only):

Acceptable

- An acceptable DSMP is provided.

Inclusion Plans

- Sex/Gender: Distribution justified scientifically
- Race/Ethnicity: Distribution justified scientifically
- Inclusion/Exclusion Based on Age: Distribution justified scientifically
- Acceptable plans for participant inclusion.

Vertebrate Animals

Not Applicable (No Vertebrate Animals)

Resubmission

- The study team has responded well to the major concerns raised in the initial review, including conducting a small pilot study of the intervention in the target population using the study clinics proposed for the trial. Results support feasibility and inform intervention adaptation for ALD patients. Sample size has been increased to address power concerns. The cost-effectiveness analysis, considered potentially premature, has been integrated into the implementation aim such that these data will still be collected and cost impacts will be explored. Prior concerns regarding timeline and human subject have been addressed.

Budget and Period of Support

Recommend as Requested

CRITIQUE 2

Significance: 2
Investigator(s): 1
Innovation: 2
Approach: 5
Environment: 2

Overall Impact:

Important focus of research in patients with ALD using mobile health technologies in a randomized controlled trial. The team is well-versed with this technology and with clinical trials in the AUD/ALD space. The focus is on abstinence over 6 months using a very high number of subjects across two centers. This remains very ambitious, has very broad and vague eligibility criteria, and conflates prior AUD research with groups of ALD patients that the teams plan to recruit. The aims include cost-effectiveness, which is premature. The settings, environment and the investigators involved are excellent.

1. Significance:

Strengths

- Important topic in an under-served population.
- Mobile health technologies interfacing with patients across the spectrum of ALD is very important to increase the reach of the technologies.

Weaknesses

- None.

2. Investigator(s):

Strengths

- Excellent investigators spanning multiple disciplines.
- Excellent environment and track record.

Weaknesses

- None.

3. Innovation:

Strengths

- Important mHealth App that has a unique discussion feature.

Weaknesses

- The trial design is not reliant on objective measures and does not give the opportunity to address objective versus subjective alcohol use in this population.

4. Approach:

Strengths

- Multi-center RCT with a good time to follow-up.
- Good preliminary data regarding subject acceptance and adherence albeit for a short duration.

- Important qualitative strategies that evaluate all stakeholders and will be very informative for future development.
- Adequate stipends have been proposed.
- Peer mentoring using patients is an important aspect that could build community.
- Pitfalls and alternatives have been considered.

Weaknesses

- The eligibility criteria are vague and expansive, which risks a mis-match in patients across both arms. Is the team only focusing on patients with cirrhosis? And if yes how do they plan to ensure similar proportion of compensated and decompensated patients are enrolled? The reviewer is cognizant of page limits so even checked the clinical trial components and these were not more granular even there.
- Since this could span patients across a wide spectrum of ALD, there is a risk of decompensation, further decompensation especially hepatic encephalopathy over this long period. These have not been accounted for.
- Alcohol use within the last 12 months is an inclusion criterion, which adds to the vagueness of the inclusion criteria.
- Smartphones although common, are by no means universal in their ownership or the ability of subjects to use them; only 10% budget is set aside for this. This may have major implications for diversity.
- This a very ambitious proposal with a huge sample size that needs to be followed for >6 months. The chances of dropout are very high and the imputation plan proposed may not be adequate to counter the missing data.
- Abstinence days at 6 months as the primary endpoint is highly fraught with issues noted with data “missingness” above; the team could have considered abstinence days at an earlier timepoint.
- Interactions with initiation/stopping of current AUD therapies in the form of Medical and behavioral ones are not adequately addressed.
- Objective criteria for alcohol use are missing and should be commented on.
- Cost-effectiveness is too premature currently.

5. Environment:

Strengths

- Excellent environment across both sites.

Weaknesses

- None.

Study Timeline:

Strengths

- Has been presented well

Weaknesses

- Is very ambitious to enroll and follow these many ALD patients over this trial duration.

Protections for Human Subjects

Acceptable Risks and/or Adequate Protections

Data and Safety Monitoring Plan (Applicable for Clinical Trials Only):

Acceptable

Inclusion Plans

- Sex/Gender: Distribution justified scientifically.
- Race/Ethnicity: Distribution justified scientifically.
- Inclusion/Exclusion Based on Age: Distribution justified scientifically.
- Very vague criteria as mentioned above.

Vertebrate Animals

Not Applicable (No Vertebrate Animals)

Biohazards

Acceptable

Resubmission

- Partially responsive to comments; the team has to be commended since they did a pilot trial which increases confidence in this design. But the N is still too high and the cost-effectiveness piece is still taking up valuable effort.

Resource Sharing Plans

Acceptable

Authentication of Key Biological and/or Chemical Resources

Acceptable

Budget and Period of Support

Recommend as Requested

CRITIQUE 3

Significance: 1
Investigator(s): 2
Innovation: 3
Approach: 5
Environment: 1

Overall Impact:

This 5-year R01 would test the CHESS mHealth app among adults receiving treatment for alcohol-associated liver disease (ALD). ALD is a live-threatening condition that often requires a liver transplant, but transplants are typically not provided unless the patient can demonstrate a period of alcohol abstinence. Alcohol cessation treatment among this difficult-to-treat population is inadequate with few options having demonstrating effectiveness. The potential for impact is therefore high if the proposal finds an effective treatment with good acceptability. Enthusiasm is dampened though by remaining issues with the approach and lack of consideration for the potential for induced alcohol withdrawal among patients while they are physically distant from adequate medical services.

1. Significance:

Strengths

- Treatment of alcohol use in ALD has very low success rates, and novel approaches are needed.
- An app, if used consistently, has the potential to overcome travel and cost barriers to accessing treatment.

Weaknesses

- Low racial and ethnic diversity of the proposed sample.

2. Investigator(s):

Strengths

- Excellent qualifications all around.

Weaknesses

- The team is large with overlapping skillsets and duplicated roles among multiple team members.

3. Innovation:

Strengths

- Test of efficacy and implementation into clinic flow in one trial.
- Incorporation of cost-effectiveness analysis.

Weaknesses

- Evaluating implementation efforts and cost-effectiveness may prove premature for an intervention with relatively unknown effectiveness in this hard-to-treat population.

4. Approach:

Strengths

- Broad inclusion/exclusion criteria to increase generalizability.

Weaknesses

- Clinic type is confounded with recruitment method. The multidisciplinary clinics will involve personal recruitment by study physicians, which could affect engagement in a way that would appear to be an effect of clinic type, affecting Hypothesis 3c.
- Four approaches to treating missing data are described in the data analysis, but it’s not indicated how the primary approach will be determined for assessing the primary hypothesis.
- There is not enough detail provided in the power analysis to determine if it is appropriate to the analyses. It appears that it was either done on a different unspecified model, or important

considerations for mixed-effects repeated-measures regression modeling (e.g., intraclass correlation, variance estimates for outcome variable) were not specified. Nor was it specified which effect (interaction term vs. planned contrast) was the focus of the power analysis, or what 0.3 sigma corresponds to in terms of number of drinking days.

- The cost effectiveness analysis describes what cost components will contribute to the analysis but doesn't describe the statistical analyses that will be used to evaluate the hypothesis.
- 6-month attrition rates are estimated at 7.5% based on a population of primary care patients. The proposed population is very different and will likely yield higher attrition rates.
- Research has shown poor recall accuracy with the TLFB beyond a few weeks back, and the proposed study will use a 90-day TLFB. It's unclear why the app couldn't be leveraged to assess alcohol use more frequently.
- No procedures to mitigate the risks of alcohol withdrawal.

5. Environment:

Strengths

- Excellent support for the proposed research.

Weaknesses

- None.

Study Timeline:

Strengths

- Acceptable.

Weaknesses

- The project timeline text in the research strategy doesn't match the project timeline figure.
- The timeline figure indicates just 3 months of startup activities.

Protections for Human Subjects

Unacceptable Risks and/or Inadequate Protections

- For a remotely delivered treatment study for individuals with heavy alcohol use, the lack of any acknowledgement of or mitigation plan for alcohol withdrawal symptoms is unacceptable.

Data and Safety Monitoring Plan (Applicable for Clinical Trials Only):

Acceptable

- An institutional DMC/DSMB will oversee the study.

Inclusion Plans

- Sex/Gender: Distribution justified scientifically.
- Race/Ethnicity: Distribution justified scientifically.
- Inclusion/Exclusion Based on Age: Distribution justified scientifically.
- All adults, genders, races, and ethnicities eligible. Distribution of these groups expected to match those with ALD at the associated clinics.

Vertebrate Animals

Not Applicable (No Vertebrate Animals)

Resubmission

- Applicants were mostly responsive to reviews. Concerns about power remain and they still didn't provide a resource sharing plan.

Budget and Period of Support

Budget Modifications Recommended (in amount/time)

Recommended budget modifications or possible overlap identified:

- The salary for Co-I M. German, M.D., is listed as under $37,000. It seems as though the base salary and salary requested values may have been transposed, which would result in the funds requested being inadequate for the role described.

THE FOLLOWING SECTIONS WERE PREPARED BY THE SCIENTIFIC REVIEW OFFICER TO SUMMARIZE THE OUTCOME OF DISCUSSIONS OF THE REVIEW COMMITTEE, OR REVIEWERS' WRITTEN CRITIQUES, ON THE FOLLOWING ISSUES:

PROTECTION OF HUMAN SUBJECTS: UNACCEPTABLE
Reviewers noted the lack of a mitigation plan for alcohol withdrawal symptoms .

INCLUSION OF WOMEN PLAN: ACCEPTABLE

INCLUSION OF MINORITIES PLAN: ACCEPTABLE

INCLUSION ACROSS THE LIFESPAN: ACCEPTABLE

COMMITTEE BUDGET RECOMMENDATIONS: The budget was recommended as requested.

Footnotes for 1R01AA030470-01A1; PI Name: Quanbeck, Andrew

Ad hoc or special section application percentiled against "Total CSR" base.

NIH has modified its policy regarding the receipt of resubmissions (amended applications).See Guide Notice NOT-OD-18-197 at https://grants.nih.gov/grants/guide/notice-files/NOT-OD-18-197.html. The impact/priority score is calculated after discussion of an application by averaging the overall scores (1-9) given by all voting reviewers on the committee and multiplying by 10. The criterion scores are submitted prior to the meeting by the individual reviewers assigned to an application, and are not discussed specifically at the review meeting or calculated into the overall impact score. Some applications also receive a percentile ranking. For details on the review process, see http://grants.nih.gov/grants/peer_review_process.htm#scoring.

MEETING ROSTER

The roster for this review meeting is displayed as an aggregated roster that includes reviewers from multiple AA Special Emphasis Panels of the NIAAA Special Emphasis Panels for the 2023/10 council round.
This roster for AA is available here.

Response Letter for R01AA030470-01A1 Review Comments

Dear Dr. Radaeva:

Our thanks to you and the review panel for their positive feedback and constructive criticism. Below you will find detailed responses to the major criticisms raised.

Sample size concerns (R2): The PI team has successfully recruited large sample sizes for several CHESS studies with loss to follow-up rates of 7.5% in a concurrent study of this platform in those with AUD (R01-AA024150). In response to concerns about feasibility due to large size (R2) and budgetary concerns, we have recalculated our sample size using a more realistic intraclass coefficient of 0.10. With 7.5% loss to follow-up and a moderate effect size estimate of 0.3 (using published effect sizes from prior CHESS platform studies), we would have power ≥80% to assess our primary hypothesis of increased abstinent days with 298 participants (149 per randomization group). This reduces the required recruitment numbers by 63 and improves feasibility of recruitment and retention as well as easing budgetary concerns.

Missing data (R1,R2): Due to concerns about missing data and loss-to-follow-up, we will change our primary endpoint to 3 months, making 6 months an exploratory endpoint, which will improve retention rates for assessing the primary outcome of days of abstinence. This change aligns with alcohol intervention literature which frequently use primary efficacy endpoints of 12 weeks for AUD interventions as well as aligning with our use of 90-day Timeline Follow-back (concern raised by R1). In addition, we will follow published best practices (Hallgren KA, et al *ACER* 2016; Witkiewitz K et al *ACER* 2014) for handling missing alcohol data in trials of AUD interventions by utilizing multiple imputation and full information maximum likelihood methodology.

Feasibility concerns (R2): To address concerns regarding feasibility, we have removed the cost-effectiveness analysis, which was felt to be premature (R1, R2), and addressed sample size concerns above (R2). Moving the primary endpoint to 3 months, with 6 months an exploratory endpoint, also enhances our feasibility to successfully recruit and retain the required number of participants.

Poorly described usual care condition (R1): In hepatology clinics, usual care for alcohol is minimal and, when it is provided at all, may involve recommendations for AUD treatment. We will use an *enhanced* usual care condition, as in Dr. Mellinger's recently funded R01AA030748, where participants will be given a brochure on alcohol treatment resources. For all participants, any AUD treatment received, whether in multidisciplinary ALD clinics or elsewhere, will be recorded at time of enrollment and all subsequent follow-up timepoints.

Inclusion criteria concerns (R1, R2): We retain any past year use of alcohol as inclusion criteria to preserve greater generalizability (as noted favorably by R3) and because lack of sustained remission (e.g., abstinence of less than one year) is associated with greater relapse rates, thus indicating a potential ongoing need for additional interventions to sustain abstinence. We retain any stage of ALD in our criteria to maintain broad generalizability and allow us to provide data on the efficacy of AUD interventions in early-stage ALD, which is a major gap in AUD/ALD intervention research. To address concerns about variable enrollment of later-stage ALD (R2), we will block randomize based on stage of ALD (early-stage [no cirrhosis] versus late-stage [cirrhosis or acute alcoholic hepatitis]) to account for the impact of ALD stage on outcomes.

Redundant roles on research team: Hepatology and alcohol use expertise is required at each site to successfully complete the study aims. However, to reduce redundancy, Dr. Lucey's role will be changed to an Other Significant Contributor without effort.

Risk of hepatic decompensation (R2): Participants will be in clinical care with their hepatologist. Any new or worsening hepatic decompensation events will be treated per standard-of-care by their medical physicians.

Lack of racial diversity in participants (R1,R3): We have translated the entire CHESS platform into Spanish and broadened our inclusion criteria to include Spanish-speaking participants. We will make every effort to enroll a racially diverse sample, allowing us to begin exploring culturally sensitive issues and modify the platform as needed for future, larger studies. If efficacy is established in this trial, future trials will include more centers, utilizing PI Mellinger's existing collaborations within the liver disease research community (e.g., New York, Texas, Florida) to test the CHESS intervention in a more racially and culturally diverse population.

Many thanks again for this opportunity. Please don't hesitate to reach out with any additional questions.

Sincerely, the CHESS-ALD Investigators (PIs Andrew Quanbeck, Randy Brown, and Jessica Mellinger)
